# MicroRNA-142 Critically Regulates Group 2 Innate Lymphoid Cell Homeostasis and Function

**DOI:** 10.4049/jimmunol.2000647

**Published:** 2021-06-01

**Authors:** Luke B. Roberts, Geraldine M. Jowett, Emily Read, Tomas Zabinski, Rita Berkachy, Murray E. Selkirk, Ian Jackson, Umar Niazi, Nelomi Anandagoda, Masatake Araki, Kimi Araki, Jagath Kasturiarachchi, Chela James, Tariq Enver, Rachael Nimmo, Rita Reis, Jane K. Howard, Joana F. Neves, Graham M. Lord

**Affiliations:** * School of Immunology and Microbial Sciences, King’s College London, London, United Kingdom;; † Centre for Host-Microbiome Interactions, King’s College London, London, United Kingdom;; ‡ Centre for Craniofacial and Regenerative Biology, King’s College London, London, United Kingdom;; § Wellcome Trust Cell Therapies and Regenerative Medicine PhD program, London, United Kingdom;; ¶ Department of Life Sciences, Imperial College London, United Kingdom;; ‖ Guy’s and St Thomas’ National Health Service Foundation Trust and King′s College London National Institute for Health Research Biomedical Research Centre Translational Bioinformatics Platform, Guy’s Hospital, London, United Kingdom;; # Institute of Resource Development and Analysis, Kumamoto University, Kumamoto, Japan;; ** University College London Cancer Institute, University College London, London, United Kingdom;; †† School of Life Course Sciences, King’s College London, London, United Kingdom; and; ‡‡ Faculty of Biology, Medicine and Health, University of Manchester, Manchester, United Kingdom

## Abstract

Innate lymphoid cells are central to the regulation of immunity at mucosal barrier sites, with group 2 innate lymphoid cells (ILC2s) being particularly important in type 2 immunity. In this study, we demonstrate that microRNA(miR)-142 plays a critical, cell-intrinsic role in the homeostasis and function of ILC2s. Mice deficient for miR-142 expression demonstrate an ILC2 progenitor–biased development in the bone marrow, and along with peripheral ILC2s at mucosal sites, these cells display a greatly altered phenotype based on surface marker expression. ILC2 proliferative and effector functions are severely dysfunctional following *Nippostrongylus brasiliensis* infection, revealing a critical role for miR-142 isoforms in ILC2-mediated immune responses. Mechanistically, *Socs1* and *Gfi1* expression are regulated by miR-142 isoforms in ILC2s, impacting ILC2 phenotypes as well as the proliferative and effector capacity of these cells. The identification of these novel pathways opens potential new avenues to modulate ILC2-dependent immune functions.

## Key Points

- MicroRNA-142 isoforms critically regulate ILC2 homeostasis and effector functions.
- MicroRNA-142 isoforms regulate the ILC2 lineage cell intrinsically.
- *Socs1* and *Gfi1* are miR-142 isoform regulated targets in ILC2s.

## Visual Abstract

**Figure:**
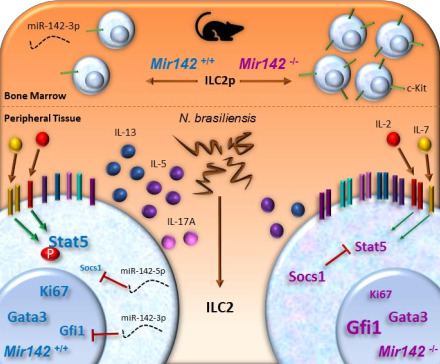

## Introduction

Innate lymphoid cells (ILCs) play critical roles in an array of biological processes, particularly at mucosal barrier sites. However, dysregulation of ILC activity also contributes to the onset of chronic inflammatory and autoimmune diseases such as inflammatory bowel disease, asthma, allergy, and atopic dermatitis (1).

ILCs can be classified into subsets, which reflect those of an αβ T cell paradigm. Group 1 ILCs include NK cells and helper-like ILC1s, which are critical innate producers of molecules such as IFN-γ and TGFβ (2). These cells are the innate equivalents of CD8^+^ T cells and CD4^+^ Th_1_ cells, respectively. Group 3 ILCs express RORγt and include lymphoid tissue inducer cells as well as NK cell receptor–expressing (NCR^+^ ILC3) or –negative (NCR^–^ILC3) subsets, which express IL-17 and IL-22, like CD4^+^ Th_17_. Group 2 ILCs (ILC2s), are reliant on a number of transcription factors, including GATA3 (3, 4), growth factor independent 1 (GFI1) (5), RORα (6) and T cell factor 1 (TCF1) (7). They express receptors for epithelial-derived cytokines including IL-33, IL-25, and among a growing number of recognized functions, contribute to type 2 immune responses via the expression of cytokines such as IL-5, IL-13, IL-4, and IL-9, similar to Th_2_ cells.

Like other helper-like ILC subsets, ILC2s are lineage-negative cells expressing IL-7Rα (CD127). These cells express an array of characteristic surface markers, including (but not limited to) Klrg1, Icos, IL-2Rα (CD25), c-Kit, and Sca-1, which aid their identification by immunophenotyping. However, the expression pattern of these markers may alter depending on the tissue in which the cells are resident (8) and may be highly dynamic in response to activation state (9). In mice for example, Klrg1 is expressed by nearly all ILC2s in the intestinal lamina propria and acts as a robust marker of ILC2s at this site, whereas expression of this marker at immunological baseline is lower in other sites such as the lung (8). Heterogeneity within the ILC2 pool has also been noted, corresponding to differences in expression profiles of phenotypic markers. For instance, in the murine lung and fat-associated lymphoid tissue, “natural” ILC2s are predominantly IL-33–responsive ILC2s that lack or have limited expression of the receptor for IL-25 (IL-25R) and demonstrate low expression of Klrg1, relative to “inflammatory” ILC2s (iILC2s) (10). iILC2s are Klrg1^hi^ cells that highly express IL-25R but lack expression of the IL-33 receptor (ST2). These cells accumulate after exposure to IL-25 and during immune responses directed by parasitic worms. iILC2 can also take on an ILC3-like profile, gaining the capacity for IL-17A expression in response to fungal infections such as *Candida albicans* (10).

Although ILC subsets functionally mirror those of αβ-T cells, the early pathways that regulate their development diverge downstream of the bone marrow (BM)–common lymphoid progenitor shared by both lineages. All murine ILCs arise from multipotent early innate lymphoid progenitors (11) and CD127^+^α4β7^+^ α-lymphoid progenitors (αLP). Differentiation of an inhibitor of DNA-binding 2–dependent common progenitor to all helper-like ILC subsets (ChILP), which has lost the potential for NK cell development, occurs downstream of these progenitor populations (12). From the ChILP, cells expressing transcription factor PLZF form the earliest fully committed helper-like ILC precursor (ILCp) populations, capable of differentiating into helper ILC1, ILC2, and ILC3 subsets, whereas PLZF^–^ ChILP give rise to CCR6^+^ lymphoid tissue inducer cell–expressing RORγt (13). Expression of programmed death receptor 1 (Pd-1) within the ChILP pool has been previously shown to faithfully correlate with expression of PLZF (14). An additional progenitor within the ILCp can be further identified, which appears to only hold potential for development into the ILC2 subset and is referred to as the ILC2 progenitor (ILC2p) (3, 14, 15).

MicroRNAs (miRs) are noncoding RNAs, which maintain cell type–specific transcriptomes by posttranscriptionally regulating gene expression. This is primarily achieved by binding of the miR seed region situated at the 5′ end of the mature miR strand, with complementary sites in the 3′ untranslated region (UTR) of the target transcript. Of the miRs found in hematopoietically derived cell types, miR-142 is particularly highly and ubiquitously expressed (16). Expression of miR-142 occurs as two mature isoforms, miR-142-3p and miR-142-5p, synthesized by differential processing of the miR-142 precursor hairpin, derived from a single, highly evolutionarily conserved locus. Within the immune system, miR-142 plays profound and nonredundant roles in multiple facets of immune cell development, fate, and function (17–22). This is concordant with reported aberrations in miR-142 expression during immune-pathologies (23–27) and with its critical role in the maintenance of peripheral immune tolerance (28, 29). Importantly, miR-142 expression in lymphocytes such as T cells has been associated with anti-inflammatory actions, with downregulation of expression observed in association with activated cell phenotypes (23, 30). Recently, it has been demonstrated that expression of miR-142 isoforms play important roles in the homeostasis of group 1 ILCs (31). However, how miR-142 regulates other helper ILC subsets is unknown.

In this study, we extend the understanding of the roles of miR-142 in immune system function by revealing that ILC2 homeostasis and function is critically dependent on cell-intrinsic expression of miR-142 isoforms.

## Materials and Methods

### Mice

*Mir142*^–/–^, *Mir142*^fl/fl^*CD4*^cre+^, and *Mir142*^fl/fl^*ER^T2^Cre* mice were generated and maintained as previously described (28, 29). The *B6-Mir142^em2Card^* strain carries a 26-bp deletion of the *miR-142-3p* sequence generated using CRISPR-Cas9 by the Centre for Animal Resources and Development, Kumamoto University. *Rag1*^–/–^ mice were bred at King’s College London and *Rag1*^–/–^*Mir142*^–/–^ mice were generated by in-house breeding of *Mir142*^–/–^ and *Rag1*^–/–^ lines. C57BL/6J wild-type (WT) mice were purchased from Charles River, U.K. *Cd*4^Cre^ mice were kindly provided by Professor Matthias Merkenschlager (Medical Research Council London Institute of Medical Sciences, Imperial College London, U.K.). Tamoxifen-inducible, Rosa26-Cre recombinase (Rosa26-Cre-ERT2)–transgenic mice were kindly provided by Dr. Thomas Ludwig (Columbia University) and generated using the Cre-ER^T2^ construct generated by Pierre Chambon at the Institute of Genetics and Molecular Biology (University of Strasberg). *Rag2*^–/–^*Il2rg*^–/–^ mice were a kind gift from the laboratory of Professor Richard Thompson (King’s College London). CD45.1^+^ congenic mice were a kind gift from the laboratory of Professor Giovanna Lombardi (King’s College London). Mice were housed in specific pathogen–free conditions. Male and female animals were used in sex-matched and age-controlled experiments. Animals were used between 6–8 wk of age unless otherwise stated. *B6-Mir142^em2Card^* mice were maintained at University College London under U.K. Home Office Project License PPL: 70/8143

### Study approval

All experiments were performed according to King’s College London and national guidelines, under a U.K. Home Office Project License (PPL:70/7869 to September 2018; P9720273E from September 2018).

### Cell lines

293T cells (American Type Culture Collection) used for reporter assays were maintained at 37°C/5% CO_2_ in DMEM supplemented with 10% FCS, 2 mM l-glutamine, 10 mM HEPES, 1× nonessential amino acids, 1 mM sodium pyruvate + 50 U/ml penicillin + 50 µg/ml streptomycin (complete DMEM [cDMEM]).

### Nippostrongylus brasiliensis infection model

*N. brasiliensis* life cycle was maintained in male Sprague–Dawley rats according to published methods (32). Infective L3 larvae were isolated from fecal cultures through a Baermann apparatus and used for s.c. infection. Four-hundred XL3 were injected per mouse into the left flank.

### In vivo tamoxifen dosing protocol

A total of 100-μg doses of tamoxifen (Sigma-Aldrich no. 156738) diluted in sunflower oil and warmed to 37°C were administered i.p. once daily on 3 consecutive d to *Mir142*^fl/fl^*ER^T2^Cre* mice. Mice were monitored daily following treatment. Successful deletion of miR-142 isoforms in peripheral lymphocyte populations using this protocol has previously been demonstrated (29).

### In vivo activation of ILC2s by recombinant IL-33

C57BL/6J female mice were injected i.p. with 500 ng of recombinant murine (rm) IL-33 (BioLegend) in 100 μl of PBS or PBS alone, once daily for 4 consecutive d. Animals were culled on day 5 and tissues harvested for ILC2 purification by FACS.

### Generation of mixed BM chimeras

*Rag2*^–/–^*Il2rg*^–/–^ hosts were sublethally irradiated with 400 centigray given as a split dose of 2 × 200 centigray 4 h apart. Twenty-four hours later, 2 × 10^6^ cells of total BM from donor mice was injected i.v. via the lateral caudal vein in 150 µl PBS. Donor cells were prepared from female congenic B6 (CD45.1^+^), WT C57BL/6J (CD45.2^+^), and *Mir142*^–/–^ (CD45.2^+^) mice, mixed in the indicated ratios. The donor mixed cells were validated as having the correct proportions of CD45.1^+^/CD45.2^+^ cells by flow cytometry. Host animals were individually housed and placed on enrofloxacin (Baytril) given in drinking water 48 h prior to irradiation and were maintained on antibiotics for 2 wk after donor cell transfer. Six weeks posttransfer, host animals were culled, and tissues harvested for analysis.

### Abs

Fluorophore-conjugated monoclonal Abs to the following mouse Ags were used: CD3 (17A2), CD5 (53-7.3), Ly-6G (Gr-1), CD19 (1D3), CD11c (N418), CD45 (30-F11), CD127 (A7R34), CD25 (PC61.5), Klrg1 (2F1), Sca-1 (D7), Icos (15F9), α4β7 (DatK-32), Flt3 (A2F10), NK1.1 (PK136), NKp46 (29A1.4), RORγt (B2D), T-bet (4B10), Gata3 (L50-823), IL-13 (eBio13A), IL-5 (TRFK5), IL-17A (TC11-18H10.1), Ki67 (SolA15), CD117 (2B8), pSTAT5(Y694), ST2 (RMST2-2), IL-25R (MUNC33), CD11b (M1/70), Siglec-F (E50-2440), NGFR (ME20.4), Thy1.1 (HIS51), CTLA4 (UC10-4B9), IL-10 (JES5-16E3), PD-1 (29F.1A12), Tigit (GIGD7), CD45.1 (A20), and CD45.2 (104). Polyclonal Abs to Gfi1 (unconjugated) and against rabbit IgG (Alexa Fluor 488 conjugated) were also used. Fc receptor blocking was carried out with anti-CD16/32. The panel of lineage markers used to identify peripheral lineage^–^ ILC in this study consisted of the above monoclonal Abs against CD3, CD5, CD19, Ly-6G, CD11c, CD11b, and Ter119. When analyzing BM ILC and progenitor populations, the panel of lineage markers consisted of the former in addition to NK1.1 in combination with a fluorophore-conjugated mouse hematopoietic lineage Ab mixture (Thermo Fisher Scientific) containing monoclonal Abs against CD3 (17A2), CD45R (B220), CD11b (M1/70), Ter-119 (Ter-119), and Ly-6G (GR-1).

### Tissue processing

Small intestinal lamina propria (siLP) and colonic lamina propria (cLP) leukocytes were isolated as previously described (33) using collagenase-D (0.5 mg/ml) and Dispase-II (1.5 mg/ml) digestion with 10 µg/ml DNAse I in HBSS without Mg^2+^ or Ca^2+^ + 2% FCS. A 40–80% Percoll gradient separation was used to isolate leukocyte enriched siLP and cLP samples. Lungs were perfused with PBS via injection into the heart prior to harvest and lung leukocytes were isolated by dicing lung tissue and digesting for 1 h at 37°C with 300 U/ml collagenase-II + 150 µg/ml DNAse I in PBS without Mg^2+^ or Ca^2+^, followed by sample mashing through a 70-µm cell strainer into single-cell suspension. BM was isolated by flushing the femur and tibia of both hind legs per animal with PBS using a 27-gauge needle on to a 40-µm cell strainer and mashing it through the membrane. Red cell removal was performed on all samples using standard ACK lysis.

### Flow cytometry and Sorting

Data were acquired using a BD LSRFortessa (BD Biosciences). Cells were sorted using a BD FACS ARIA III (BD Biosciences) with the 355-nm laser turned off. Only samples with a purity above 97% were used. Data analysis was carried out using Flowjo software (TreeStar).

### In vitro alarmin activation of ILC2s

siLP ILC2s were FACS purified from C57BL/6J female mice. Samples were split and cultured in cDMEM with combinations of either recombinant human (rh) IL-7 + rhIL-2 (10 ng/ml each) or rhIL-7 + rhIL-2 + rmIL-33 + rmIL-25 (10 ng/ml each) (BioLegend) for 24 h at 37°C/5% CO_2_ in U-bottom 96-well plates. Following this, cells were recovered, washed twice in ice cold PBS, and lysed in Qiazol (Qiagen) at –80°C for later use.

### Intracellular cytokine capture and exhaustion marker analysis

Intracellular cytokine capture for flow cytometric detection of IL-13 and IL-5 was carried out by restimulating samples with 50 ng of PMA (Sigma-Aldrich), 1 µM ionomycin (Sigma-Aldrich) in the presence of 1 µM monensin for 4 h in cDMEM at 37°C/5% CO_2 ._ Unstimulated controls were cultured in cDMEM with monensin only. Following viability and surface marker staining, cells were fixed and permeabilized with the Foxp3 Staining Kit (eBiosciences). For analysis of exhaustion markers, including IL-10, samples were stimulated with 100 ng of PMA, 2 µM ionomycin for 3 h in the presence of 1 µM monensin, and 1× brefeldin-A (eBioscience) in cDMEM at 37°C/5% CO_2_. Unstimulated samples were cultured with monensin and brefeldin-A only. Fixation was carried out for 10 min with methanol-free 2% paraformaldehyde followed by permeabilization using the Foxp3 permeabilization buffer (eBiosciences). Pd-1 and Tigit were carried out as extracellular surface stains and Ctla4 was incorporated as both an extracellular and intracellular stain.

### STAT5 phosphorylation assay

Lamina propria leukocytes were isolated as described and rested for 1 h at 37°C/5% CO_2_ in cDMEM prior to use. Prior to stimulation, samples were stained for ILC2 and NCR^+^ ILC markers (CD45, lineage markers, Klrg1, Sca-1, Icos, NK1.1, NKp46) using fluorophore-conjugated Abs. CD127, typically used for ILC identification, was omitted to avoid the potential for preactivation of IL-7Rα signaling pathways. Samples were stimulated with rhIL-7 (10 ng/ml) or rhIL-2 (10 ng/ml) or left unstimulated. Viability staining was carried out 10 min prior to fixation. Samples were fixed at the desired time point with prewarmed 4% formaldehyde for 10 min. Samples were permeabilized with prechilled 100% methanol at –20°C for 30 min. Overnight intracellular staining for pSTAT5(Y694) was carried out at 4°C in PBS.

### Quantitative PCR

FACS purified cells were sorted directly into QIAZOL (Qiagen), vortexed, and frozen immediately on dry ice. Total lung tissue was washed in PBS and placed directly into RLT buffer (Qiagen). Gut tissue was opened longitudinally, scraped with a glass slide to remove mucus, and washed briefly in PBS then placed in RLT buffer + 10 μM/ml β-mercaptoethanol (Sigma-Aldrich). Total RNA was isolated using the RNeasy Micro Kit or miRNeasy Micro Kit (Qiagen), according to the manufacturer’s instruction. mRNA was reverse transcribed to cDNA using random hexamers and the RevertAid First Strand cDNA Synthesis Kit (Thermo Fisher Scientific). miRs were amplified and reverse transcribed using the TaqMan Advanced miRNA cDNA Synthesis Kit. Reactions for detection of *Socs1*, *Gfi1*, *Il33,* miR-142-3p, miR-142-5p, *Gapdh*, and small nuclear RNA (snRNA) U6 were carried out in 10 µl volumes using 2X Maxima probe/ROX qPCR Master Mix (Thermo Fisher Scientific) and TaqMan real-time quantitative PCR (qPCR) assays with FAM-labeled probes were performed. Reactions for detection of *Dclk1*, *Il25*, and *Gapdh* were carried out in 10 μl volumes using Fast SYBR-Green Mix (Applied Biosystems) Master Mix. Assays were analyzed with an ABI Prism 7900HT Real-Time PCR Instrument or ViiA 7 Real-Time PCR System (Applied Biosystems), where the Ct values were extracted. Relative gene expression and fold changes were calculated using the 2^–ΔΔCt^ method.

### miR-142–target gene reporter assay

Reporter and miR expression plasmid constructs were designed and generated as previously described (28). The 239T cells were plated into 12-well plates at 7.3 × 10^4^ cells per well, 24 h before transfection, and 3.28 ng of pcDNA-tNGFR-poly(A)-PGK-Thy1.1 reporter plasmid containing either the WT or mutated *Mus musculus Gfi1* or *Gata3* 3′ UTR and 1.17 μg pMY-miR-142-IRES-PAC or pMY-IRES-PAC control plasmid was cotransfected into each well using polyethyleneimine in quadruplicate. Reporter expression was determined 48 h after transfection by staining the cells with anti-NGFR and anti-Thy1.1 followed by staining with DAPI for LIVE/DEAD cell discrimination. Data are expressed as the ratio between the mean fluorescence intensity (MFI) values obtained for CD271 (NGFR) and the Thy1.1 MFI values followed by averaging of the quadruplicate measurements. The reporter expression values from samples transfected with the miR-142 expression vector were normalized to the values of the samples transfected with the empty expression vector, which was set to 1.

### RNA sequencing

RNA was extracted from c-Kit^+^Lin^–^Sca1^+^ (KLS) BM cells, and RNA-sequencing libraries were prepared using SMART-Seq v4 Ultra Low Input RNA Kit for Sequencing (634891, Takara Bio, Kusatsu, Japan), and the Nextera XT Library Preparation Kit (FC-131-1096; Illumina, San Diego, CA). Libraries were then sequenced on an Illumina NextSeq 550 using the 150 Cycle High Output v2 Kit (FC-404-2002) with 2 × 75–bp read length.

### Bioinformatics analysis

#### Raw data to count matrix

ShortRead and Trimmomatic were used for FASTQ file quality checks and to remove sequencing adapters. The FASTQ files were aligned to mouse genome GRCm38/mm10 using Hisat2, whereas SAMtools was used to remove low quality alignments and duplicates. Number of reads aligned to each gene were counted using Genomic Alignments.

#### Exploratory data analysis and filtering

The data matrix (*p* genes, *n* samples) was filtered for genes with mean of less than 3 to remove very low count genes. Using low dimensional summaries of the data matrix (e.g., principal components), the samples showed stratification with batches 1 and 2, hence the data in each batch were normalized separately and the batch identifier was adjusted for at the modeling level.

#### Model description

The expression for each gene was modeled as a negative binomial variable, where the average difference between *B6-Mir142^em2Card^* (miR-142 knockout) and WT groups in each batch 1 and 2 were estimated as coefficients using a varying intercepts hierarchical modeling framework.

#### Pathway analysis

All analysis was implemented in R and Stan. Model fit checks were performed using posterior predictive simulations. Pathway overrepresentation and gene set enrichment analysis were performed using GOStats and Generally Applicable Gene-set Enrichment.

The analysis source code is as follows: https://github.com/uhkniazi/BRC_ILC_Joana_PID_21.

### Statistical analysis

Normality testing was conducted using the Shapiro–Wilk test. Parametric data were analyzed using two-tailed, *t* test with Welch correction. Mann–Whitney *U* test was performed on nonparametric data. Unless otherwise stated, the mean and SEM are represented in data visualization. Experiments were not carried out under blinded conditions. Statistics analysis was carried out in Prism v.8.0. Significance levels are expressed as *p* values where *p < 0.05, **p < 0.01, ***p < 0.001, and ****p < 0.0001.

## Results

### BM ILC2 progenitors are phenotypically altered and more prevalent in the absence of miR-142

miR-142 is highly expressed by cells of the hematopoietic lineage, including hematopoietic stem cells which are often studied by isolating the c-Kit^+^ (Kit^+^) lineage^–^ Sca-1^+^ population (KLS) found in the BM. However, the KLS population is only enriched for hematopoietic stem cells and contains other cell types. Following analysis of RNA-sequencing data generated from the KLS population of mice with a genomic CRISPR-mediated deletion of miR-142 (*B6-Mir142^em2Card^*), our model predicted 118 genes to be differentially overexpressed and 101 genes to be underexpressed in miR-142–deficient samples compared with controls; these gene sets were enriched for gene ontology terms related to numerous aspects of immune system regulation and function (Supplemental Fig. 1A–C). Interestingly, markers of the ILC2 lineage were among the predicted overexpressed transcripts (Supplemental Fig. 1D) among other genes reported to contribute to an ILC2-like transcriptional signature (Supplemental Fig. 1E) (14, 34–39). Therefore, our data suggest that the absence of miR-142 might lead to alterations to ILC2 development from BM ILC precursor populations.

**FIGURE 1.**
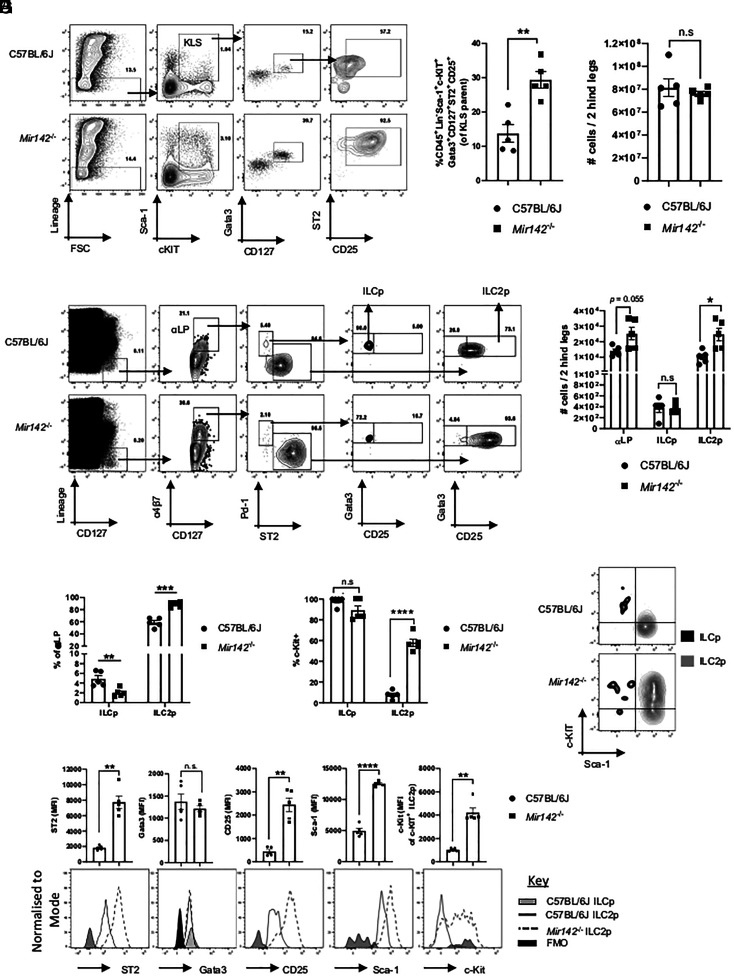
BM ILC2 progenitors are phenotypically altered and more prevalent in the absence of miR-142. (**A**) Representative flow cytometry plots demonstrating an enriched pool of CD45^+^Lineage^–^Sca1^+^c-Kit^+^Gata3^+^CD127^+^CD25^+^ST2^+^ cells within the BM KLS (c-Kit^+^Lineage^–^Sca-1^+^) population of *Mir142*^–/–^ mice compared with C57BL/6J (WT) controls. (**B**) Summary of proportion (%) of KLS population expressing the CD45^+^Lineage^–^Sca1^+^c-Kit^+^Gata3^+^CD127^+^CD25^+^ST2^+^ phenotype. (**C**) Total BM cell counts from both hind legs of WT and *Mir142*^–/–^ mice. (**D**) Gating protocol for identification of αLP (Lineage-CD127+a4B7^hi^), ILC precursors (ILCp: Lineage^–^CD127+a4B7^hi^ST2^–^Pd-1^+^Gata3^+^CD25^–^), and ILC2p (Lineage-CD127+a4B7^hi^ST2^+^Pd-1^+/–^Gata3^+^CD25^+^) within the BM pool of WT and *Mir142*^–/–^ mice. Plots are pregated on live, single, CD45^+^, nonautofluorescent cells. (**E**) Total αLP, ILCp, and ILC2p cell counts per two hind legs of individual mice. (**F**) Percentage of ILCp and ILC2p populations within the αLP pool. (**G**) Summary quantification of c-Kit expression by ILCp and ILC2p in WT and *Mir142*^–/–^ mice. (**H**) Representative flow cytometry plot comparative overlays for c-Kit and Sca-1 expression by ILCp and ILC2p in WT and *Mir142*^–/–^ mice. (**I**) Summary quantification of ILC2p marker MFI (top) and representative histogram overlays for expression of ST2, Gata3, CD25, Sca-1, and c-KIT by ILC2p across genotypes (bottom). Marker MFIs are for total cells within the ILC2p gate, except for c-Kit MFI, which is the MFI of c-KIT^+^ ILC2p. In histograms, WT ILCp are shown as controls for ILC2p marker expression. For Gata3, a fluorescence minus one (FMO) gating control is shown. Data shown are representative of three similar, independent experiments with *n* = 3–5 age and sex matched mice per genotype. Gate numbers represent the percent of the indicated parental gate. *p < 0.05, **p < 0.01, ***p < 0.001, ****p < 0.0001. n.s., nonsignificant.

Using mice with a germline deletion of the entire *Mir142* locus (*Mir142*^–/–^) (29) we confirmed relative enrichment of ILC2-like cells within the *Mir142*^–/–^ BM KLS pool (Fig. 1A, 1B). We next investigated BM ILC precursors, including ILCp and ILC2p populations found within the αLP progenitor population. There were no differences in BM total cell number (Fig. 1C) or number of αLP progenitor cells (Fig. 1D, 1E) between the genotypes. Likewise, no alterations were found to the number of the ILCp precursor population (Lineage^–^CD127^+^α4β7^+^ST2^–^Pd-1^+^Gata3^+^CD25^–^) (Fig. 1D, 1E). However, the proportion of ILCp within the αLP population was decreased in *Mir142*^–/–^ mice in favor of an enhanced proportion of cells with an ILC2p phenotype (Lineage^–^CD127^+^α4β7^+^ST2^+^Pd-1^+/–^Gata3^+^CD25^+^) (Fig. 1F), and the number of ILC2p were increased overall (Fig. 1E). ILCp from both genotypes were also positive for c-KIT and negative for Sca-1, as previously described (40), whereas *Mir142*^–/–^ ILC2p demonstrated greatly enhanced expression of c-KIT, despite most WT ILC2p being c-KIT^–/lo^ as expected (Fig. 1G, 1H) (3, 14, 15). This explained why an enhanced population of ILC2-like cells were found to be present within the KLS population of *Mir142* deficient mice but not WT animals. Interestingly, expression of surface markers for *Mir142*^–/–^ ILC2p were greatly enhanced compared with WT ILC2p overall, despite comparable Gata3 expression between the genotypes (Fig. 1I).

Based on these data, we conclude that isoforms of miR-142 play critical roles in defining the phenotype of ILC2p in the BM and may play important roles in restricting the developmental program of the ILC2p/ILC2 lineage.

### miR-142 controls ILC2 homeostasis at peripheral mucosal tissue sites

**FIGURE 2.**
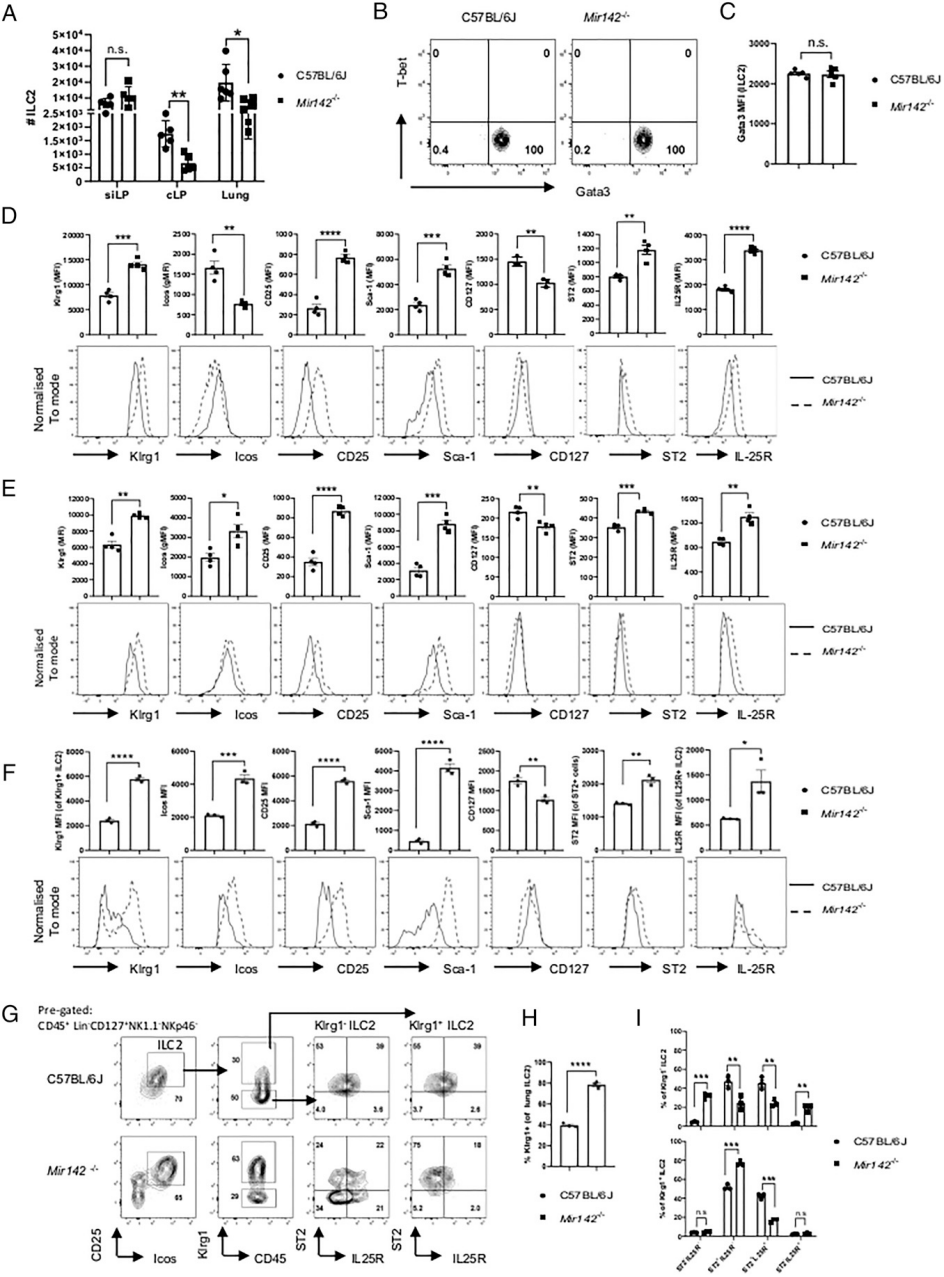
Peripheral ILC2 homeostasis at mucosal sites is critically regulated by miR-142. (**A**) Total number of ILC2s in the siLP, cLP, and lungs of *Mir142*^–/–^ and C57BL/6J (WT) mice. (**B**) Representative flow cytometry plots of Gata3 and T-bet staining in siLP ILC2s. Quadrant numbers indicate percent of the ILC2 parent population. (**C**) Summary of Gata3 staining MFI of WT and *Mir142*^–/–^ lung ILC2s. (**D**) Representative histograms (top) and summary MFI (bottom) of ILC2 phenotypic markers including ICOS, CD25, Sca-1, CD127, Klrg1, ST2, and IL-25R for WT and *Mir142*^–/–^ siLP ILC2s. (**E**) As for (D) but for cLP ILC2s. (**F**) As for (D) but for lung ILC2s. (**G**) Differential analysis of ST2 and IL-25R expression by Klrg1^–^ and Klrg1^+^ lung ILC2, identified as CD45^+^Lineage^–^CD127^+^NK1.1-NKp46-CD25^+^Icos^+^ cells in C57BL/6J and *Mir142*^–/–^ mice. Gate numbers present percentage of parental gate. (**H**) Percentage of lung ILC2s expressing Klrg1. (**I**) Quantification of data represented in (G) for percentage of total Klrg1^–^ (top) and Klrg1^+^ (bottom) lung ILC2s expressing combinations of ST2 and IL-25R. Graphs depict mean ± SEM. (A and B) Welch *t* test and (D) unpaired *t* test. *p < 0.05, **p < 0.01, ***p < 0.001, ****p < 0.0001. Data are representative of three independent experiments with between *n* = 3–5 age- and sex-matched mice per genotype. n.s., nonsignificant difference.

In peripheral tissues, ILC2s are enriched at mucosal barrier sites including siLP, cLP, and lung. In *Mir142*^–/–^ mice, ILC2 numbers were unchanged in the siLP, whereas in the cLP and lung, there were fewer ILC2s (Fig. 2A, Supplemental Fig. 2A, 2B). These findings were robust in the face of different gating methods used to identify ILC2s (Supplemental Fig. 2C, 2D). *Mir142*^–/–^ ILC2s retained high levels of Gata3 expression, without coexpression of T-bet (Fig. 2B, 2C) or RORγt (Supplemental Fig. 2C), supporting their identity as ILC2s. After analysis ex vivo, a striking observation was that *Mir142*^–/–^ ILC2s in these peripheral sites displayed a vastly altered cell surface phenotype compared with WT controls, matching observations in BM *Mir142*^–/–^ ILC2p (Fig. 2D–F, Supplemental Fig. 2E). This phenotype was largely characterized by enhanced surface expression of numerous ILC2 markers including CD25, Sca-1, Klrg1, and alarmin receptors ST2 (IL-33R) and IL-25R, whereas CD127 was downregulated compared with controls. An exception was a reduced expression of Icos by siLP *Mir142*^–/–^ILC2s (Fig. 2D), despite significant overexpression of Icos by cLP and lung *Mir142*^–/–^ ILC2s (Fig. 2E, 2F, Supplemental Fig. 2E). None of the 3′UTR regions of the transcripts for these markers were identified as direct targets of miR-142 isoforms (DIANA microT-CDS, TargetScanMouse v7.1). Increase of these markers on ILC2s was therefore not likely to be explained by direct gene derepression in the absence of miR-142.

Interestingly, although Klrg1 is not highly expressed by lung ILC2s at immunological baseline (8), the majority of ILC2s in *Mir142*^–/–^ lungs were Klrg1^+^ (Fig. 2G, 2H) and significantly overexpressed Klrg1 compared with control Klrg1^+^ ILC2s (Fig. 2F). Given the alterations to alarmin receptor expression, along with an enhanced expression of Klrg1, we therefore questioned whether the ILC2 phenotype of *Mir142*^–/–^ mice could be explained by a preferential increase in iILC2s. However, differential analysis of the Klrg1^–^ and Klrg1^+^ lung ILC2 populations revealed that the predominant IL-25R-expressing population in *Mir142*^–/–^ mice was Klrg1^–^ ILC2s, not Klrg1^+^ cells (Fig. 2G, 2I). Additionally, the IL-25R^+^ST2^–^ ILC2 population observed only in *Mir142*^–/–^ lungs was found only in the Klrg1^–^ population; Klrg1^+^ ILC2s were highly ST2^+^, whereas iILC2s are reported to be ST2^–/lo^. Therefore, the effects on ILC2s observed in these mice were not obviously explained by a selective expansion of conventional Klrg1^hi^ or IL-25R^+^ iILC2s.

### miR-142–deficient ILC2s are defective in response to *N. brasiliensis* infection

Given the altered phenotypic profile of *Mir142*^–/–^ ILC2s, we investigated whether ILC2 effector functions would also be altered under conditions known to activate the cells in vivo. Damage by tissue migrating helminths at barrier sites is a critical inducer of alarmin release from epithelial cells and ILC2s are established as central innate effectors of the antihelminth type 2 immune response. Therefore, we used *N. brasiliensis* infection as a model to probe these responses in vivo.

**FIGURE 3.**
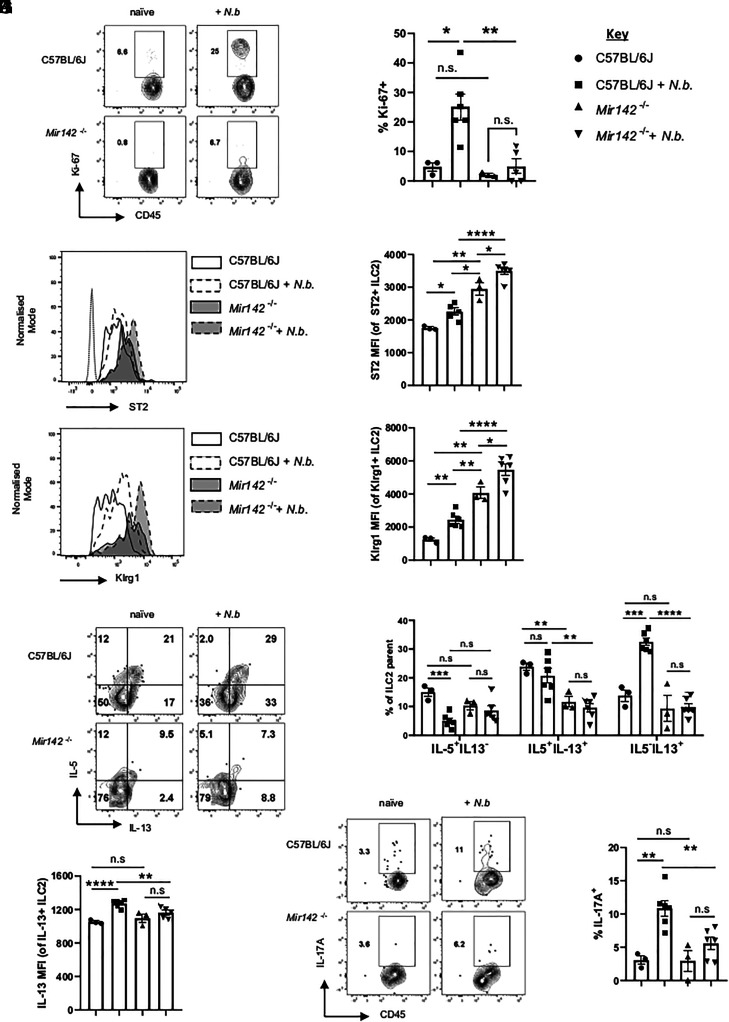
miR-142–deficient ILC2s are defective in response to *N. brasiliensis* infection. (**A**) Representative flow cytometry plots of lung ILC2s Ki-67 staining, directly ex vivo. Gate numbers represent the proportion (%) of ILC2s that are Ki67^+^. (**B**) Summary for percent of lung ILC2s expressing Ki-67. (**C**) Representative histogram overlay of ST2 staining on lung ST2^+^ ILC2s. ST2 Fluorescence minus one (FMO) staining control is indicated by the dotted line. (**D**) MFI of ST2 staining on total ST2^+^ lung ILC2s. (**E**) As for (C) but for Klrg1 staining. (**F**) As for (D) but for Klrg1 MFI. (**G**) Representative flow cytometry plots for IL-5 and IL-13 expression by lung ILC2s following sample restimulation ex vivo. Quadrant numbers represent the percent of the ILC2s parental gate. (**H**) Percent of lung ILC2s expressing combinations of IL-5 and IL-13. (**I**) IL-13 MFI of total IL-13^+^ ILC2s. (**J**) Percent of lung ILC2s expressing IL-17A. (**K**) Summary for percent of lung ILC2s expressing IL-17A. *n* = 6 sex- and age-matched mice for *N. brasiliensis*–infected groups and *n* = 3 sex- and age-matched mice for naive (uninfected) groups. Analysis of cells from infected animals was done at day 5 postinfection. *p < 0.05, **p < 0.01, ***p < 0.001, ****p < 0.0001. n.s., nonsignificant difference.

Adult worms were found in the small intestine of all infected mice. Parasite burden of the small intestine at day 5 postinfection was not significantly different between *Mir142*^–/–^ and WT genotypes. (Supplemental Fig. 3A). The total number of CD45^+^ leukocytes in the lungs of *Mir142^–/–^* mice was significantly lower than in control lungs (Supplemental Fig. 3B). ILC2 numbers in the lung were not altered by *N. brasiliensis* infection at the time point investigated in either genotype (Supplemental Fig. 3C). However, proliferative responses of *Mir142^–/–^* ILC2s were significantly impaired following infection compared with WT ILC2s from infected mice, which significantly upregulated Ki-67 compared with ILC2s from infection naive WT lungs (Fig. 3A, 3B). ST2 and IL-25R expression were enhanced on WT ILC2s in response to infection, as expected (Fig. 3C, 3D, Supplemental Fig. 3D, 3E). However, despite the elevated expression of these receptors by *Mir142*^–/–^ ILC2s from naive mice, ST2 expression was further enhanced by *Mir142*^–/–^ ILC2s in response to infection (Fig. 3C, 3D) as was Klrg1 (Fig. 3E, 3F). This was not the case for IL-25R (Supplemental Fig. 3D, 3E). Examination of ILC2 effector cytokine responses revealed that WT ILC2s highly expressed both IL-5 and IL-13 (Fig. 3G, 3H, Supplemental Fig. 3F) and significantly upregulated the amount of IL-13 produced following *N. brasiliensis* infection (Fig. 3I). However, *Mir142*^–/–^ ILC2s were inhibited from effector cytokine production at both baseline and in response to infection (Fig. 3G–I). This was not in favor of an enhanced production of IL-17A, which was also lacking in *Mir142*^–/–^ mice (Fig. 3J, 3K). Recently, a Runx-dependent exhaustion phenotype for ILC2s has been described, characterized by a lack of responsiveness to alarmin signaling (41). However, analysis of Pd-1, Tigit, IL-10, and Ctla4 expression by lung ILC2s revealed no obvious differences between genotypes (Supplemental Fig. 3G, 3H), suggesting that a prior exhausted state does not underlie nonresponsiveness of lung *Mir142*^–/–^ ILC2s to *N. brasiliensis* infection.

These data reveal that because of defective effector activity, *Mir142*^–/–^ ILC2s are unable to appropriately respond to parasitic helminth infection in vivo. Expression of miR-142 isoforms by ILC2s is therefore an essential component of the mechanics of the innate arm of type 2 immunity.

### Cell-intrinsic expression of miR-142 isoforms critically regulates ILC2 phenotype and function

**FIGURE 4.**
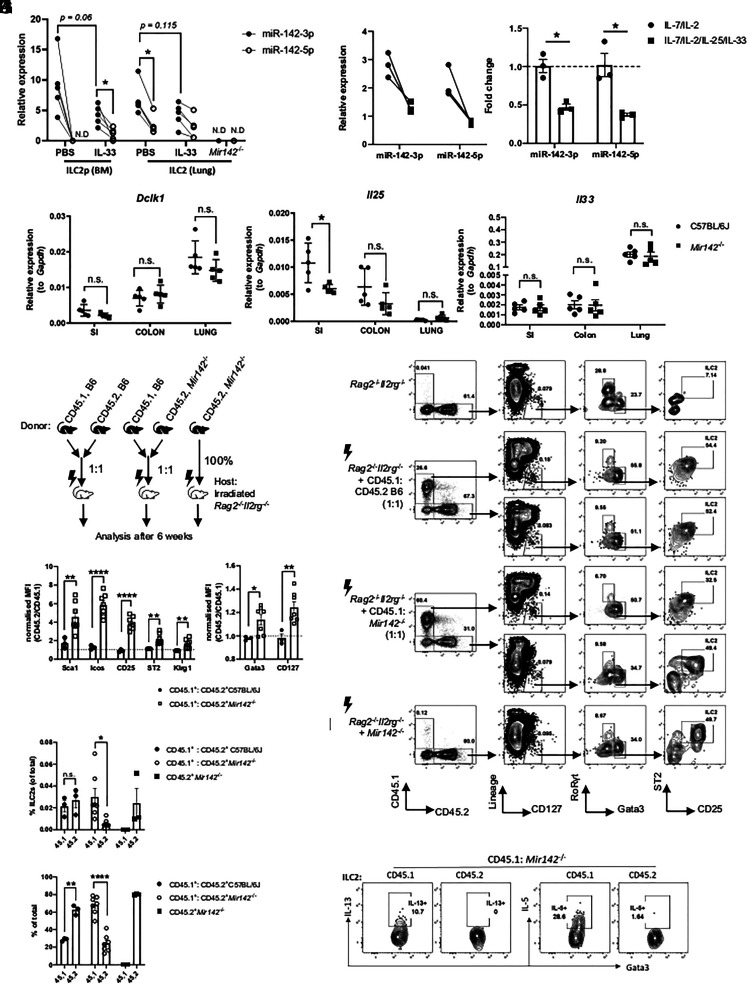
Cell-intrinsic expression of miR-142 isoforms critically regulates ILC2 phenotype and function. (**A**) Expressions of miR-142-3p and miR-142-5p (relative to snRNA U6) in BM ILC2p and lung ILC2s of female C57BL/6J mice treated with PBS or rmIL-33. Joined data points indicate values from the same samples. (**B**, left) Expressions of miR-142-3p and miR-142-5p (relative to snRNA U6) in siLP ILC2s cultured for 24 h in vitro with recombinant IL-2 + IL-7 only, or IL-2 + IL-7 + IL-25 + IL-33. Joined data points indicate paired samples from cells isolated from individual mice. (B, right) Fold change of miR-142 expression levels in IL-2 + IL-7 + IL-25 + IL-33–treated ILC2s normalized to values of IL-2 + IL-7–treated cells. (**C**) Expression of *Dclk1*, *Il25*, and *Il33*, relative to *Gapdh* expression, determined by qPCR using cDNA from total RNA samples extracted from tissue lysates prepared from small intestine (SI), colon, and lung samples from C57BL/6J (WT) and *Mir142^–/–^* mice. Animals were age- and sex-matched, *n* = 5 per genotype, and size and physiological location of each tissue sample was standardized between biological replicates and across genotypes. (**D**) Schematic detailing experimental setup of mixed BM chimera generation in irradiated (black lightning bolt) *Rag2*^–/–^*Il2rg*^–/–^ host mice. (**E**) Gating for lung ILC2s in nonirradiated CD45.2^+^ *Rag2*^–/–^*Il2rg*^–/–^ mice. (**F**) As for (E) but for irradiated *Rag2*^–/–^*Il2rg*^–/–^ host mice receiving mixed total BM from congenic CD45.1^+^ B6 and CD45.2^+^ C57BL/6J donors. (**G**) As for (F) but donor cells were from CD45.1^+^ B6 and CD45.2^+^ *Mir142*^–/–^ mice. (**H**) As for (F) but donor cells were from CD45.2^+^ *Mir142*^–/–^ mice only. (**I**) Summary of flow cytometry staining MFI values for CD45.2^+^ ILC2s expression of indicated markers, normalized to congenic CD45.1^+^ ILC2s within the same host. (**J**) CD45.1^+^ and CD45.2^+^ ILC2s expressed as proportions of total, live, single lung cells from *Rag2*^–/–^*Il2rg*^–/–^ hosts receiving the indicated donor cell mixtures. (**K**) As for (J) but for proportion of total CD45.1^+^ and CD45.2^+^ cells. (L) Representative flow cytometry plots for expression of IL-13 and IL-5 by lung CD45.1^+^ B6 (WT) and CD45.2^+^ *Mir142*^–/–^ ILC2s from *Rag2*^–/–^*Il2rg*^–/–^ hosts receiving mixed total BM of the two genotypes. *n* = 5–7 mice per experiment. *p < 0.05, **p < 0.01, ****p < 0.0001. n.s. nonsignificant.

To evaluate whether absence of cell-intrinsic miR-142 expression in miR-142–deficient ILC2s was responsible for our observations, we purified lung ILC2s and BM ILC2p from WT mice and analyzed expression of miR-142-3p and miR-142-5p isoforms by qPCR (Fig. 4A). Cells were isolated from animals at steady state (treated with PBS) and from IL-33–treated mice, as a useful model of ILC2 activation in vivo. Expression of miR-142-3p and miR-142-5p isoforms was observed in mature lung ILC2s from both PBS and IL-33–treated mice, and BM ILC2p following IL-33 treatment. Only miR-142-3p expression was observed in BM ILC2p from PBS-treated mice (Fig. 4A). Overall, miR-142-3p appeared to be slightly more abundant than miR-142-5p in ILC2p and lung ILC2s. A small, nonsignificant trend toward reduced miR-142-3p expression was apparent following IL-33 treatment. Both miR-142-3p and miR-142-5p were also detected in siLP ILC2s (Fig. 4B). Following short term in vitro culture (24 h) in the presence of IL-2, IL-7, IL-25, IL-33, and miR-142 isoform expression levels were reduced compared with cells cultured in IL-2 and IL-7 alone (Fig. 4B).

Given the upregulation of markers associated with activation state of the cells (9) by *Mir142^–^*^/–^ ILC2s and ILC2p (Fig. 1, Fig. 2), we questioned whether stimuli responsible for steady-state activation of the cells, such as IL-25 (42) might be enhanced at mucosal sites in these mice. However, *Mir142^–/–^* mucosal tissues did not express greater transcript abundance for *Il25* or the Tuft cell marker *Dclk1* (Fig. 4C). Expression of transcripts for IL-33 was also unaltered between genotypes (Fig. 4C). Next, to determine whether the altered peripheral ILC2 phenotype in *Mir142*^–/–^ mice was solely developmentally programmed, we used a tamoxifen (TXN)-inducible model of *Mir142* deletion (*Mir142*^fl/fl^*ER^T2^Cre*) (29). Prior to TXN treatment, both control (*Mir142*^fl/fl^*ER^T2^Cre*^WT/WT^) and *Mir142*^fl/fl^*ER^T2^Cre*^WT/+^ adult littermates had the same composition of ILC subsets and ILC phenotypes, as expected (data not shown). However, following TXN administration, peripheral *Mir142*^fl/fl^*ER^T2^Cre*^WT/+^ ILC2s displayed an altered phenotype equivalent to *Mir142*^–/–^ ILC2s (Supplemental Fig. 4A, 4B). This suggests that in WT ILC2s, miR-142 isoforms actively maintain the ILC2 state in the periphery. To address whether this disruption of ILC2 homeostasis was dependent on miR-142 expression in lymphocytes known to interact with ILCs (43, 44), we used mice with *Mir142* deletion only in T cells (*Mir142*^fl/fl^*Cd4*^Cre+^). ILC2s in these mice were normal with no evidence of the alterations displayed in either constitutive or temporal models of miR-142 deletion (Supplemental Fig. 4C, 4D). We next asked whether the observed phenotype was because deletion of miR-142 solely in the innate immune compartment. *Rag1*^–/–^*Mir142*^–/–^ retained the altered ILC2 phenotype observed in *Mir142*^–/–^ mice (Supplemental Fig. 4E, 4F).

To further probe the case for cell-intrinsic regulation of ILC2s by miR-142 isoforms, we generated mixed BM chimeras by transferring mixtures of congenic (CD45.1^+^) B6, WT (CD45.2^+^) C57BL/6J, and *Mir142*^–/–^ (CD45.2^+^) BM into irradiated alymphoid *Rag2*^–/–+^*Il2rg*^–/–^ hosts as indicated (Fig. 4D). Adoptive transfer of *Mir142*^–/–^ BM only, was carried out as a control for development of the *Mir142*^–/–^ ILC2 phenotype in the absence of competing WT cells. *Rag2*^–/–^*Il2rg*^–/–^ are widely referred to as “ILC deficient.” However, few studies acknowledge that this strain does retain a small number of residual CD45.2^+^Lineage^–^CD127^+^, immature ILC-like cells, some of which are ILC2-like cells based on Gata3^hi^-staining expression and absence of RORγt expression (Fig. 4E). However, the absence of markers of ILC2 maturity such as ST2 and CD25 (Fig. 4E) enabled sufficient gating of mature donor ILC2s in animals receiving BM transfers (Fig. 4F–H). In hosts receiving CD45.1^+^ B6/CD45.2^+^ B6 cells (the WT control donor group), the phenotype of CD45.1^+^ and CD45.2^+^ ILC2s was equivalent 6 wk posttransfer, as judged by analyzing normalized marker staining MFIs (CD45.2/CD45.1) (Fig. 4F, 4I). In contrast, despite the presence of CD45.1^+^ WT cells, CD45.2^+^ ILC2s in animals receiving CD45.1^+^ B6/CD45.2^+^ *Mir142*^–/–^-mixed BM displayed a highly altered phenotypic profile based on marker staining MFI (Fig. 4G, 4I). This phenotype was comparable with ILC2s, which developed in hosts receiving *Mir142*^–/–^ BM only (Fig. 4H). Interestingly, CD45.2^+^ *Mir142*^–/–^ ILC2s in hosts receiving CD45.1^+^ B6/CD45.2^+^ *Mir142*^–/–^ donor cells demonstrated slightly elevated expression of Gata3 and CD127, comparative with congenic CD45.1^+^ ILC2s (Fig. 4I). It is possible that these observations reflected homeostatic selection processes related to survival of *Mir142*^–/–^ ILC2s in the presence of competing WT cells. In hosts receiving CD45.1^+^ B6/CD45.2^+^ B6 BM, the proportion of CD45.1^+^ and CD45.2^+^ ILC2s within the total samples were equivalent (Fig. 4J). This was despite a significant bias for engraftment of CD45.2^+^ total cells overall (Fig. 4K); a recognized phenomenon in mixed CD45.1^+^/CD45.2^+^ reconstitution experiments with WT genotypes (45). However, in hosts receiving CD45.1^+^ B6/CD45.2^+^ *Mir142*^–/–^-mixed cells, the expected CD45.2^+^ engraftment bias was reversed in favor of CD45.1^+^ cells (Fig. 4K), indicating that *Mir142*^–/–^ cells display severe disadvantages in either their development or the process of engraftment, in the presence of *Mir142* replete competitors. This observation was also reflected by a significantly lower proportion of CD45.2^+^ *Mir142*^–/–^ ILC2s overall, compared with CD45.1^+^ WT control cells (Fig. 4J). Importantly, despite enhanced Gata3 expression, CD45.2^+^ *Mir142*^–/–^ ILC2s from mixed chimeras displayed severely restricted expression of type 2 effector cytokines, despite good expression by CD45.1^+^ WT ILC2s when stimulated with PMA/ionomycin ex vivo (Fig. 4L).

These data demonstrate that ILC2s and ILC2ps express mature miR-142 isoforms in mice, and strongly support the hypothesis that these regulatory molecules play critical, cell-intrinsic roles in establishing cellular phenotypes and effector functions of the ILC2 lineage.

### Optimal γ-chain cytokine signaling is mediated by miR-142-5p repression of Socs1 in ILC2s

**FIGURE 5.**
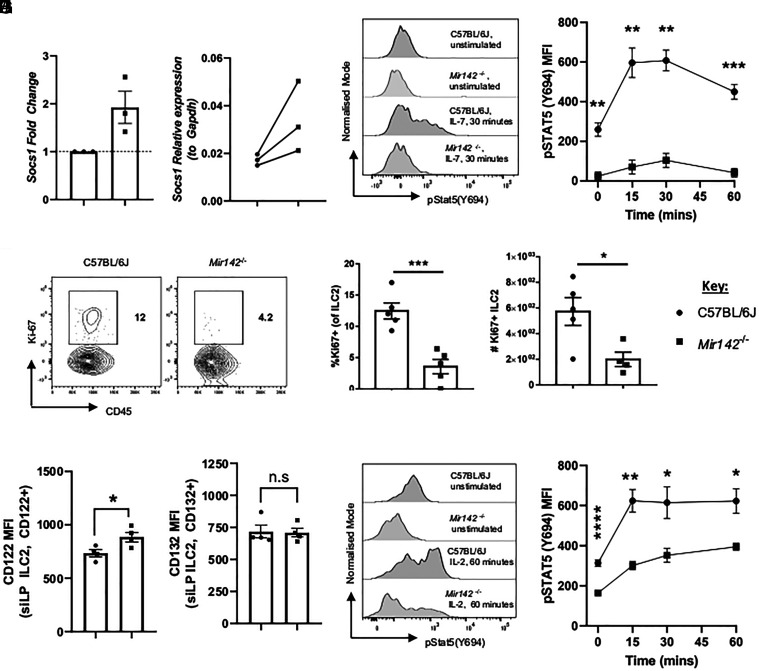
Optimal γ-chain cytokine signaling is mediated by the repression of *Socs1* by miR-142-5p in ILC2s. (**A**) Expression of *Socs1* relative to *Gapdh* in ILC2s (CD45^+^lineage^–^CD127^+^NK1.1^–^NKp46^–^Klrg1^+^Sca-1^+^) from siLP. (**B**) Fold change of *Socs1* in *Mir142*^–/–^ compared with WT ILC2s for data from (A). (**C**) Representative histograms for pStat5(Y694) induction by either unstimulated siLP ILC2s, or siLP ILC2s following ex vivo exposure to IL-7 (10 ng/ml) for 30 min. (**D**) Summary data for pStat5(Y694) induction by either unstimulated siLP ILC2s, or siLP ILC2s following ex vivo exposure to IL-7 for the time points indicated. Zero minutes indicates unstimulated samples. (**E**) Representative flow cytometry plots for Ki-67 staining in siLP ILC2s. Gate numbers represent percentage of parental ILC2 gate. (**F**, left) Percent of Ki-67^+^ ILC2s as in (E) and (F, right) enumeration of Ki-67^+^ siLP ILC2s as in (E). (**G**) MFI of CD122 (IL-2Rβ) staining on siLP ILC2s. (**H**) as for (G) but for CD132 (IL-2Rγ) staining. (**I**) As for (C) but following ex vivo exposure to IL-2 (10 ng/ml) for 60 min. (**J**) As for (D) but following ex vivo exposure to IL-2 for the time points indicated. Data (A and B) represent samples from three independent experiments of pooled biological siLP replicates in which *n* = between 3–5 mice per independent repeat. Paired *Mir142*^–/–^ and WT samples from each independent experiment are indicated by connected data points. Data (C–J) carried out with between *n* = 3–5 sex- and age-matched mice per genotype and/or treatment group. Data (E–G) representative of results of two independent experiments. *p < 0.05, **p < 0.01, ***p < 0.001, ****p < 0.0001. n.s. nonsignificant.

*Socs1* is a reported miR-142-5p target (25, 46, 47) and Socs1 overexpression in ILC2s has previously been associated with defective cytokine production (48). Indeed, *Socs1* transcript levels were increased. In *Mir142*^–/–^ ILC2s, consistent with a recent report investigating the role of miR-142 in ILC1s (31) (Fig. 5A, 5B). Socs1 inhibits Jak/Stat signaling pathways and correspondingly, *Mir142*^–/–^ ILC2s demonstrated significantly lower induction of pStat5(Y694) following ex vivo stimulation with IL-7, compared with controls (Fig. 5C, 5D). IL-7 signaling via Stat5 plays a critical role in maintenance of peripheral ILC2 populations and interestingly, lower pStat5 was also apparent in unstimulated *Mir142*^–/–^ ILC2s (Fig. 5C, 5D). This may explain the observation that *Mir142*^–/–^ ILC2s demonstrated significantly reduced Ki-67 expression, indicating a reduced proliferative capacity of the cells (Fig. 3A, 3B, Fig. 5E, 5F). However, IL-7Rα (CD127) is also decreased on *Mir142*^–/–^ ILC2s (Fig. 2) and might underlie reduced Stat5 activation. Therefore, we also assessed the capacity of *Mir142*^–/–^ ILC2s to respond to IL-2 stimulation, as *Mir142*^–/–^ ILC2s and progenitors overexpress the IL-2R subunit CD25 (IL-2Rα) (Fig. 1I, Fig. 2D–F). However, they were not deficient for CD122 (IL-2Rβ) (Fig. 5G) or CD132 (IL-2Rγ) (Fig. 5H). Nonetheless, whereas control siLP ILC2s demonstrated a rapid and robust Stat5 phosphorylation in response to IL-2 stimulation ex vivo, responsiveness to IL-2 was significantly inhibited in *Mir142*^–/–^ ILC2s (Fig. 5I, 5J). These data indicate that γc-cytokine signaling in *Mir142*^–/–^ ILC2s is defective. A possible explanation for this is that under normal settings, miR-142-5p acts to restrict *Socs1* expression, to support ILC2 homeostasis and proliferation at baseline.

### Growth factor independent 1 is regulated by miR-142-3p in ILC2s

**FIGURE 6.**
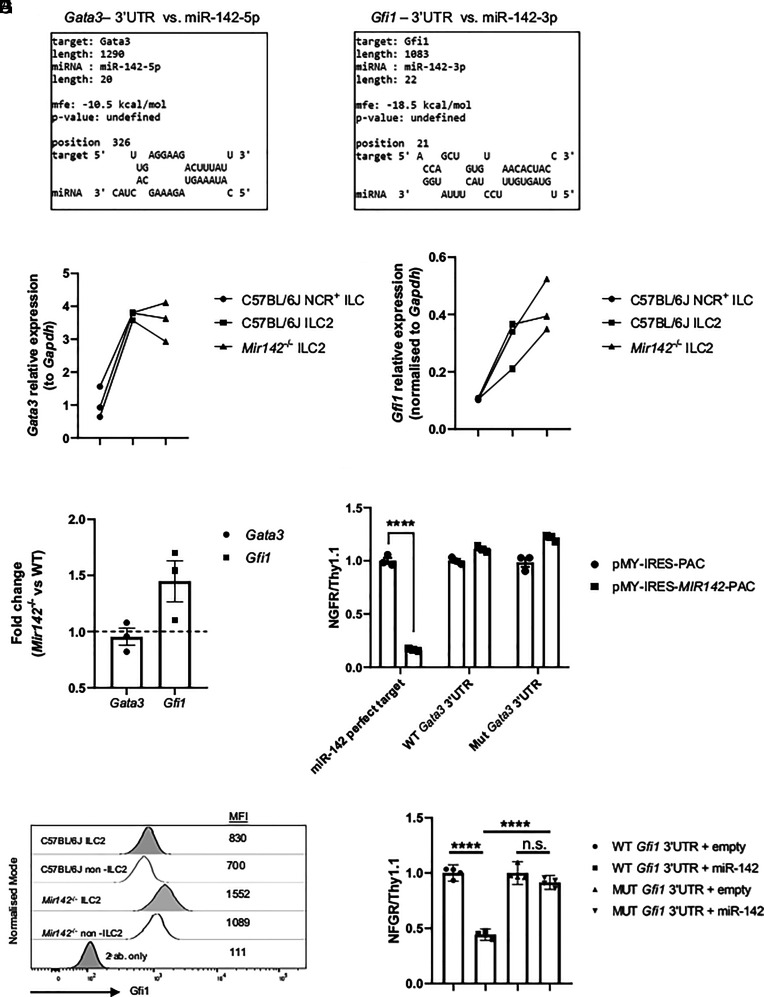
*Gata3* is not targeted by miR-142; however, *Gfi1* is a target of miR-142-3p in ILC2s. (**A**) In silico identification of a predicted miR-142-5p target site in the 3′UTR of the *Mus musculus Gata3* mRNA transcript. Schematic from RNAhybrid analysis used to determine the minimum free energy (mfe) for the interaction between the *Gata3* 3′UTR predicted target site and miR-142-5p. (**B**) As for (A) but for the predicted target site between miR-142-3p and the 3′UTR of *Mus musculus Gfi1.* (**C**) Expression of *Gata3* relative to *Gapdh* in FACS purified ILC2s (CD45^+^Lineage^–^CD127^+^NK1.1^–^NKp46^–^Klrg1^+^Sca-1^+^) isolated from the siLP of C57BL/6J (WT) and *Mir142^–/–^* mice. *Gata3*-relative expression of purified siLP NCR^+^ ILCs (CD45^+^Lineage^–^CD127^+^NK1.1^+^NKp46^+^) from WT mice was included as a biological control for the assay. (**D**) As for (C) but for *Gfi1.* (**E**) Data from (C) and (D) expressed as *Mir142*^–/–^ ILC2 *Gata3* or *Gfi1* fold change using WT values to normalize expression. (**F**) miR-142 isoforms do not directly target the predicated *Gata3* seed site identified in (A). Expression of an NGFR reporter gene that has either the WT or a mutated (Mut) *Gata3* 3′ UTR miR-142-5p binding site inserted in the presence of an empty control (pMY-IRES-PAC) or miR-142 expression vector (pMY-IRES*-Mir14*2-PAC) in HEK293T cells. A vector with a perfect target site for *Mir142*-mediated knockdown (miR-142 perfect target) was used as a positive control for miR-142–mediated repression in the assay. (**G**) Representative histograms for siLP ILC2s (CD45^+^Lineage^–^CD127^+^NK1.1^–^NKp46^+^Klrg1^+^Sca-1^+^) and non-ILC2 ILCs (CD45^+^Lineage^–^CD127^+^Klrg1^–^) staining with rabbit anti-mouse anti-Gfi1 followed by secondary staining with Alexa Fluor 488–conjugated goat anti-rabbit IgG. MFI values for each population are indicated. A secondary Ab only sample (omitting rabbit anti-mouse anti-Gfi1 staining) was the negative staining control. (**H**) Targeting of WT *Gfi1* mRNA 3′UTR by miR-142 in HEK293T cells. Mutation (Mut) of the miR-142-3p seed site of *Gfi1* prevents miR-142–mediated reporter knockdown. Data in (C)–(E): samples from three independent experiments of pooled biological siLP replicates in which *n* = between 3–5 mice per independent repeat. Paired *Mir142*^–/–^ and *Mir142*^+/+^ samples from each independent experiment are indicated by connected data points. Data in (F) and (H): two independent experiments and *n* = 4 replicates per construct, per treatment group. Paired data points represent samples analyzed from independent experiment runs. Bar graphs depict mean ± SEM. Student *t* test, *p < 0.05, ****p < 0.0001. n.s., nonsignificant.

Enhanced expression of Socs1 by *Mir142*^–/–^ ILC2s could account for the proliferative and effector defects observed. However, it could not easily account for the altered phenotypic profile of ILC2s or the augmentation in ILC2p numbers in the absence of *Mir142*. We therefore considered whether essential transcription factors relevant to ILC2 development and activation might be critically regulated by miR-142 isoforms. Among the transcription factors known to play critical roles in ILC2 development and function, we identified putative 3′UTR targets for miR-142-5p and miR-142-3p in *Gata3* (3, 4) and *Gfi1* (5), respectively (Fig. 6A, 6B). The minimum free energy value determined using RNAhybrid (49) was quite poor for Gata3 and miR-142-5p (–10.5 kcal/mol), predicting a relatively weak secondary structure for the interaction (Fig. 6A), whereas the interaction between *Gfi1* and miR-142-3p was predicted to be much more stable (–18.5 kcal/mol) (Fig. 6B). Additionally, analysis of a published AGO2-CLIP dataset identifies *Gfi1* as a potential target of miR-142-3p in lymphocytes (50). Correspondingly, *Gata3* expression in purified siLP ILC2s was not enhanced in *Mir142*^–/–^ ILC2s relative to WT ILC2s, whereas *Gfi1* demonstrated increased expression levels (Fig. 6C–E). Results of a flow cytometric reporter assay for the identification of miR-142 targets (28), also revealed that the putative miR-142-5p site in the *Gata3* 3′UTR was not a true target site (Fig. 6F). This was supported by the prior demonstration of comparable levels of Gata3 expression in ILC2s and ILC2p between genotypes (Fig. 1I, Fig. 2B, 2C). In contrast, expression of Gfi1 was greater in *Mir142*^–/–^ ILC2 relative to WT controls (Fig. 6H) and reporter assay analysis, including seed-site targeted mutagenesis, supported the putative *Gfi1* binding site as a valid target of miR-142-3p.

## Discussion

In this study, we reveal critical, cell-intrinsic roles for miR-142 isoforms in the regulation of ILC2 homeostasis and function. Previously, miR-155, miR-146a, and the miR-17∼92 cluster have also been shown to play important roles in ILC2 function (48, 51, 52). These include protection from apoptosis and expansion of ILC2s during chronic and acute airway allergen exposure, in addition to regulating ILC2 proliferation and expression of effector cytokines and Gata3 expression following exposure to IL-33. Interestingly, comparison of the miR transcriptome for Th_2_ and ILC2s demonstrates that although a large amount of overlap occurs between these cell types, a significant degree of difference is also apparent (48). miR-142 is highly expressed in T cells and its constitutive ablation results in a defect in peripheral T cell numbers through an as yet unknown mechanism (18, 19), putatively related to cell cycling defects in thymocyte precursors. In contrast, our data indicate that although the absence of miR-142 results in ILC2 deficiency in sites such as the lung and cLP (alongside a general reduction in CD45^+^ leukocyte numbers in peripheral tissues, as seen in the lung; Supplemental Fig. 3B), ILC2 numbers in the siLP are unaffected, perhaps suggestive of differential regulatory actions of miR-142 in ILC2s compared with T cells.

It is possible that the combination of a biased production of ILC2p in *Mir142*^–/–^ animals, coupled with the suggestion that ILC2p preferentially migrate to the intestinal lamina propria during the steady state (3) may underlie this unexpected observation. BM ILC2p already express the MAdCAM-1 receptor, α4β7 integrin, as well as CCR9, which is important for homing to the small intestine, which selectively expresses the CCR9 ligand CCL25. A severely restricted responsiveness to homeostatic survival factors such as IL-7 and IL-2 (likely underling dysfunctional baseline proliferative responses of ILC2s in *Mir142*^–/–^ mice) may then result in comparable overall numbers of the cells at this site with WT controls. This is despite a theoretically greater influx of cells in *Mir142*^–/–^ mice. Further work is required to understand this observation fully. *Mir142*-deficient ILC2s may serve as a useful model to investigate the nature of ILC trafficking, as well as the maintenance of mature populations of ILC2s in the periphery.

Deletion of *Mir142* leads to accumulation of ILC2s with an altered phenotype. Analysis of BM ILC2p indicates that in *Mir142*^–/–^ mice, the altered phenotype of peripheral ILC2s may be developmental in origin because of enhanced expression of markers such as CD25 and ST2. However, data from temporally deleted *Mir142*^fl/fl^*ER^T2^Cre*^WT/+^ animals suggested that miR-142 also regulates this phenotype postdevelopmentally in the periphery. Therefore, miR-142 isoform expression may be critical both during development and for homeostatic maintenance of ILC2s. It is possible that the altered phenotype of *Mir142*^–/–^ ILC2s may be related to a heightened sensitivity to tissue signals such as IL-33 and IL-25 in vivo. This could be because of either directly mediated enhanced expression of miR-142-3p target *Gfi1*, which positively regulates these receptors (5), or indirect effects of Gfi1 on Gata3 protein stability or activity (53). This hypothesis is consistent with previous studies identifying a role for IL-33 and IL-25 in the steady-state activation and function of ILC2s at tissue specific sites (42, 54, 55). Our findings suggest that miR-142 regulates aspects of the phenotype of ILC2s associated with their activation state. This is consistent with the reported anti-inflammatory regulatory actions of miR-142 in other lymphocyte lineages (23, 30) and with the indication of a negative correlation between ILC2 exposure to activation stimuli (i.e., alarmins) and miR-142 isoform expression levels. Inducible deletion of miR-142 from mature, peripheral ILC2 populations also indicates that ILC2 responses can be modulated postdevelopmentally via the manipulation of miR-142 expression. This information is potentially highly relevant in the various settings in which the modulation of ILC2 responses might be an attractive therapeutic target, such as in parasite infections, allergic disease, or asthma. More recently, attention has also turned to understanding the presence of ILC2s within the setting of the tumor microenvironment, and investigation of whether the roles played by these cells during antitumor immunity may be harnessed or repurposed therapeutically (56). Although further work will be required to investigate this prospect, manipulation of miR-142 isoforms and/or miR-142–regulated pathways in ILC2s may hold promise for development of novel immunotherapeutics benefiting the field of immuno-oncology.

Despite an altered cell surface phenotype reminiscent of an enhanced activation state, *Mir142*^–/–^ ILC2s were severely defective in their proliferative and effector responses during *N. brasiliensis* infection, as well as at baseline. Analysis of exhaustion and inhibitory checkpoint receptors (41, 57) did not suggest that *Mir142*^–/–^ ILC2s present an advanced state of cellular exhaustion overall. It is possible that high-level expression of Klrg1 by miR-142–deficient ILC2s may have contributed to the nonresponsiveness of these cells in vivo, as in human skin ILC2s, the interaction between Klrg1 and its ligand E-Cadherin is thought to be inhibitory (58). In addition, *Mir142*^–/–^ ILC2s did not appear to selectively represent iILC2s, which are known to potently produce cytokines such as IL-13 and IL-17A following activation with IL-25 and also highly express Klrg1 (10). This suggests that increased expression of Klrg1 alone does not sufficiently explain ILC2 nonresponsiveness to in vivo activation stimuli. Alternatively, regulation of *Socs1* by miR-142-5p may have critical relevance with regards to dysregulated ILC2 effector function. Previously it was shown that upregulation of *Socs1* following miR-19 deletion was partially responsible for the inhibition of ILC2 type 2 cytokine production (48) and a similar function for miR-19–regulated *Socs1* has also been reported for Th_2_ cells (59). Additionally, the reliance of ILC2s, and other ILC subsets, on the expression and function of Stat5 for the optimal expression of signature cytokine production, and the role of Stat5 in the activation of the transcription of type 2 cytokine loci have been described (60, 61). Socs1 is a critical inhibitor of Stat5 activation and signaling, and as *Mir142*^–/–^ ILC2s clearly demonstrate a profound defect in Stat5 activation mediated via γc-cytokine signals, this would explain why *Mir142^–/–^* are defective in IL-13 and IL-5 cytokine production. Additionally, Stat5 signaling mediated by IL-7 and/or IL-2 is known to mediate ILC2 homeostatic accumulation (60). Overexpression of Socs1 may therefore underlie the proliferative defects demonstrated by *Mir142*^–/–^ ILC2s, but further investigation is required to fully confirm these hypotheses.

This study indicates that *Gfi1* and *Socs1* are direct targets of miR-142 isoforms in ILC2s. Interestingly, Gfi1 is already preferentially expressed highly in ILC2s. Therefore, one interpretation of our model may be that miR142-3p regulates optimal expression of Gfi1 by ILC2s during homeostasis. This may be sufficient to direct the appropriate pathways of the ILC2 developmental program, as well as to restrict over expression of alarmin receptors and maintain the resting state of ILC2s prior to alarmin induced ILC2 activation. That miR-142-3p appeared to be the most highly expressed miR-142 isoform in ILC2ps at baseline may also suggest that regulation of optimal Gfi1 expression during ILC2 development is particularly important for correct and appropriate differentiation of this lineage.

These findings reveal novel, to our knowledge, critical molecular pathways which govern the homeostasis and functional activity of ILC2s. These pathways have the potential to be manipulated and exploited for therapeutic gain.

## Supplementary Material

Data Supplement
