# Supplementary material for: MicroRNA-142 Critically Regulates Group 2 Innate Lymphoid Cell Homeostasis and Function
Source: J Immunol. 2021 Jun 1;206(11):2725–39. doi: 10.4049/jimmunol.2000647 (PMC7610861; doi:10.4049/jimmunol.2000647)
Supplement: Data Supplement [file JI_2000647.zip › JI_2000647_Supplemental_1.pdf]

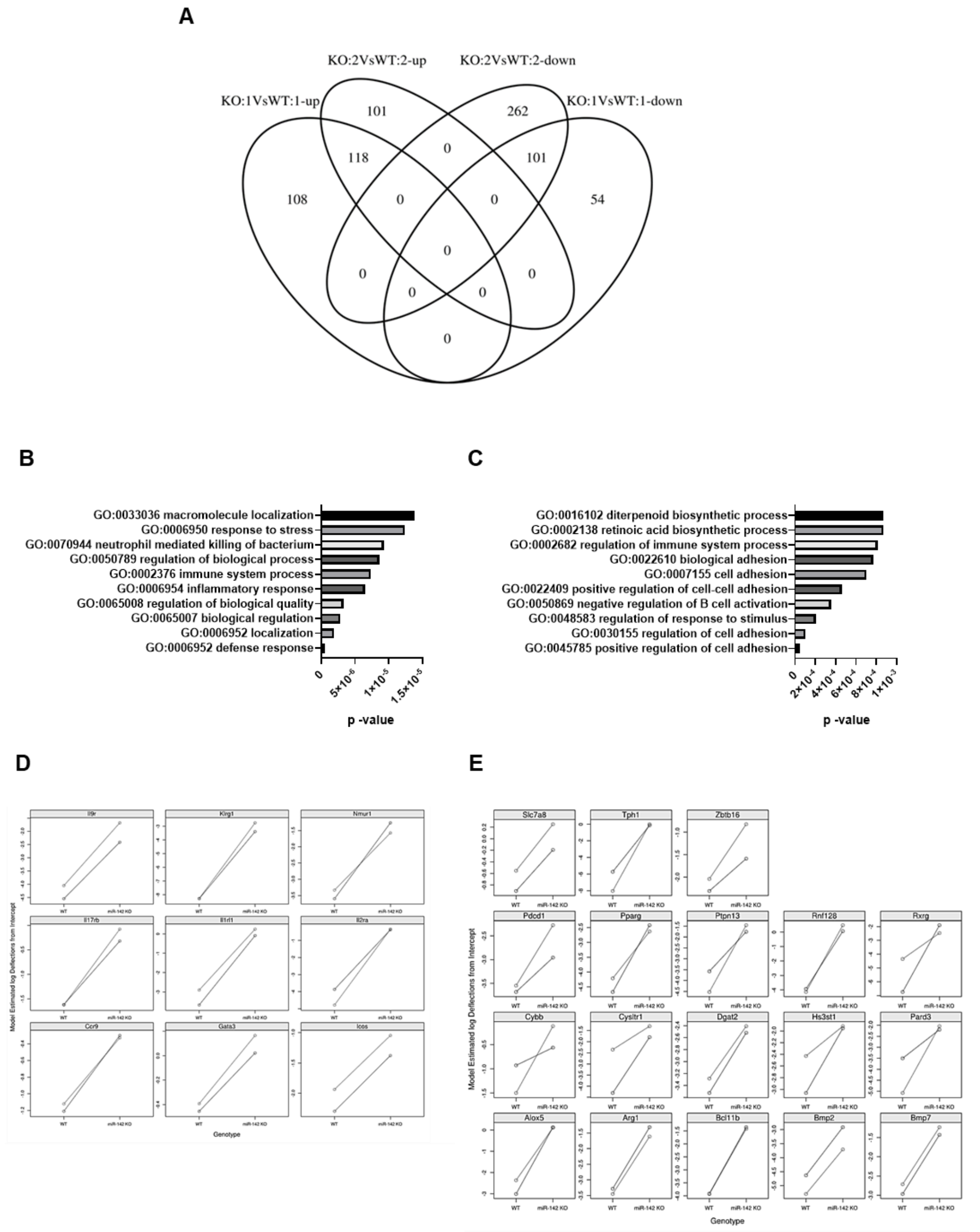

Supplemental Figure 1. Model predicted enhanced expression of ILC2 signature genes within KLS samples of genomic CRISPR-mediated miR-142 deficient mice compared to WT controls (related to Figure 1). **(A)** Venn diagram showing overlaps of differentially expressed genes up-regulated and down regulated between genotypes within two independent RNA sequencing batch runs (batch 1: KO1 + WT1, batch 2: KO2+WT2); Top 10 statistically significant GO terms from pathway over-representation analysis of up-regulated **(B)** genes in KO samples observed in both batch runs (118 genes) and down-regulated **(C)** genes in KO samples observed in both batch runs (101 genes). **(D-E)** Each panel shows the model estimated expression of a selected gene in each genotype and in each sequencing batch, represented as average log difference from model intercept (or grand mean). **(D)** Genes often used in combination to identify an ILC2-like phenotype in immunophenotyping studies. **(E)** Genes previously associated with an ILC2-like transcriptional signature (see main text body for references). Samples were prepared from bone marrow c-Kit^{+}Lineage^{-}Sca-1^{+} (KLS) populations. miR-142-KO refers to samples from *B6-Mir142^{em2Card}* mice with a genomic CRISPR-mediated deletion of the 3p-seed site of the *Mir142* gene. WT refers to *Mir142* replete controls. Samples prepared and acquired in 2 independent sequencing batch runs where n=2 biologically independent samples prepared from each genotype. Data from paired batch runs are identified by conjoined data points.

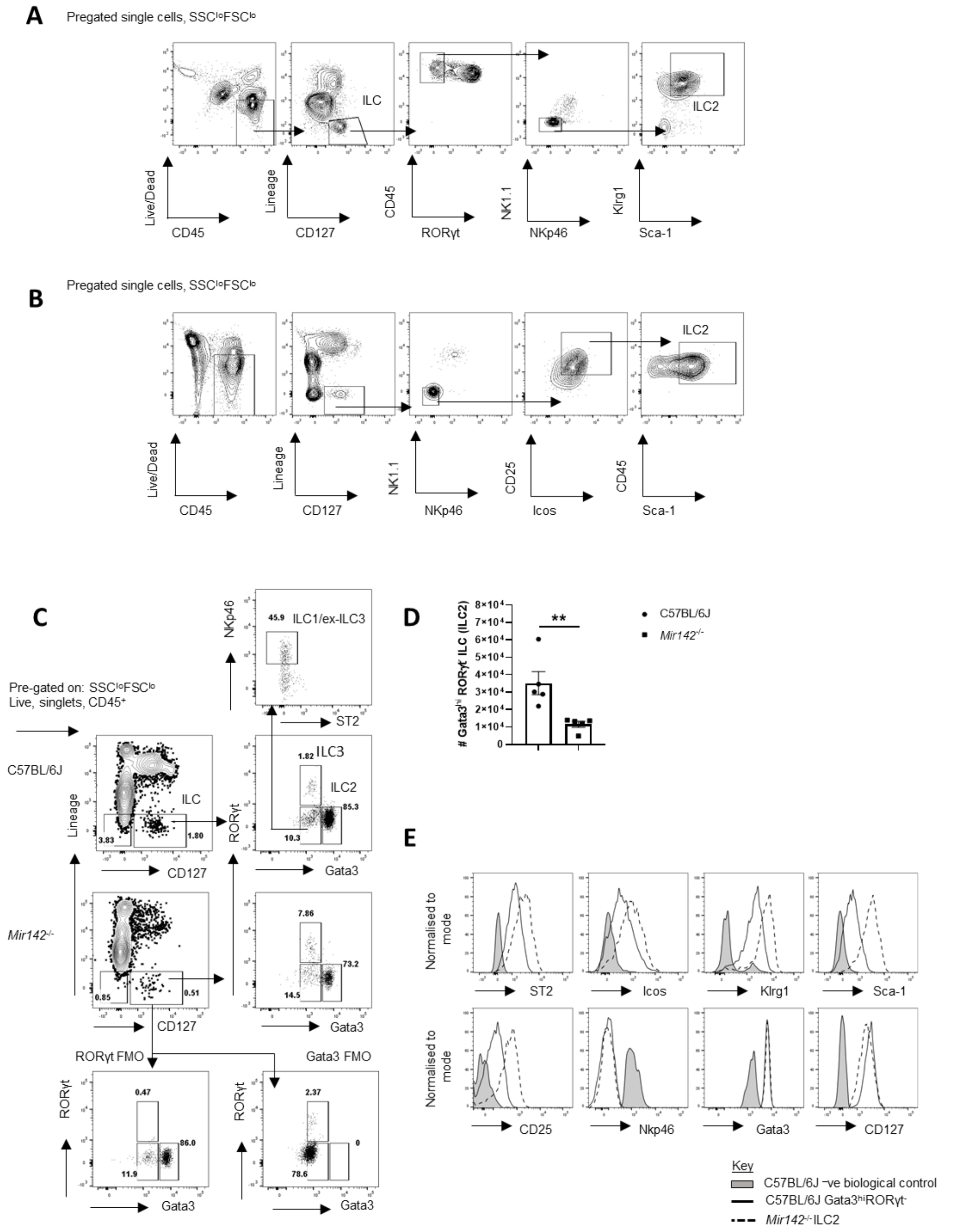

Supplemental Figure 2. Gating strategies for identification of ILC2s (related to Figure 2 and throughout manuscript). (A). Representative gating strategy for identification of ILC2 in the small intestinal and colonic lamina propria. **(B)** Representative gating strategy for identification of ILC2 in the lung. **(C)** alternative representative gating strategy for identification of ILC2s, shown her for lung ILC2s, gated as CD45+, Lineage-CD127+Gata3^{hi}Rorgt^{-} cells. **(D)** Quantification of ILC2 number in lungs of C57BL/6J (WT) and *Mir142$^{-/-}$* mice at baseline, using identification of ILC2s as shown in (C). **(E)** Representative histogram overlays of ILC2 markers (ST2, CD25, Klrg1, Sca-1, Icos, Gata3), ILC markers (CD127) and non-ILC2 markers (NKp46) in lung ILC2s from WT and *Mir142*-/- mice at baseline. Filled in histograms are biological controls for marker expression gated on either WT ILC1/ex ILC3 as shown in (C) (ST2, CD25, Klrg1, Sca-1, Icos, Gata3, NKp46) or WT Lineage-CD127- cells (CD127). Data (D-E) = n= 5 mice per genotype. **p = <0.01.

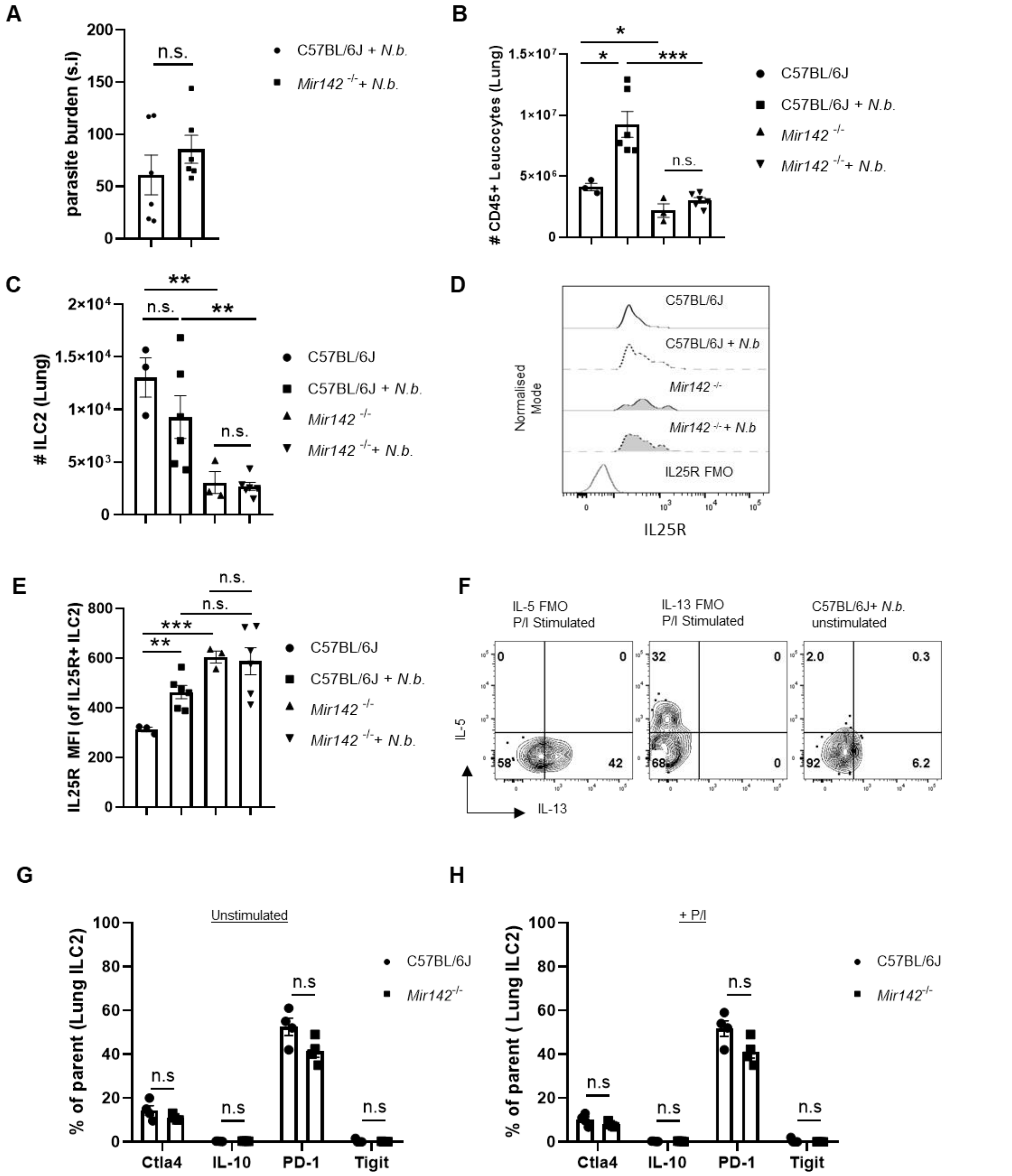

Supplemental Figure 3. ILC2 responses during parasitic roundworm infection are affected by the absence of miR-142 (related to Figure 3). (A) Small intestine *Nippostrongylus brasiliensis* (*N.b.*) parasite burden at day 5 post infection (p.i.) **(B)** Total number of $CD45^+$ leucocytes in the lungs of naïve and *N.b.* infected *Mir142*$^{-/-}$ and C57BL/6J (WT) mice. **(C)** ILC2 ($CD45^+Lineage^-CD127^+NK1.1^-NKp46^-$ $CD25^+ICOS^+$) numbers in lungs. **(D)** Representative histograms of IL25R staining on lung $IL25R^+$ ILC2. IL25R fluorescence minus one control (FMO) is Indicated. **(E)** MFI of IL25R staining on total $IL25R^+$ lung ILC2. **(F)** Gating controls for IL-5 and IL-13 staining in PMA/Ionomycin re-stimulated lung ILC2 (related to Figure 4 I-J). Gates were set based on IL-5 and IL-13 FMO controls. Lack of IL-5/IL-13 expression in WT ILC2 from *N.b.* Infected mice, not re-stimulated with PMA/Ionomycin (Unstimulated) demonstrates success of PMA/Ionomycin re-stimulation methods. **(G)** Summary of MFI values for Ctla4, IL-10, Pd-1 and Tigit, taken from flow cytometry staining of WT and *Mir142*$^{-/-}$ lung ILC2s at baseline, after *in vitro* culture of total lung cells in single cell suspension in the presence of monensin for 4 hours (unstimulated). **(H)** As for (G) but cells were cultured with PMA/Ionomycin + monensin. Bar graphs depict mean $\pm$ SEM. Unpaired t-test. *$p < 0.05$, **$p < 0.01$, ***$p < 0.001$, ****$p < 0.0001$. n.s., non-significant difference

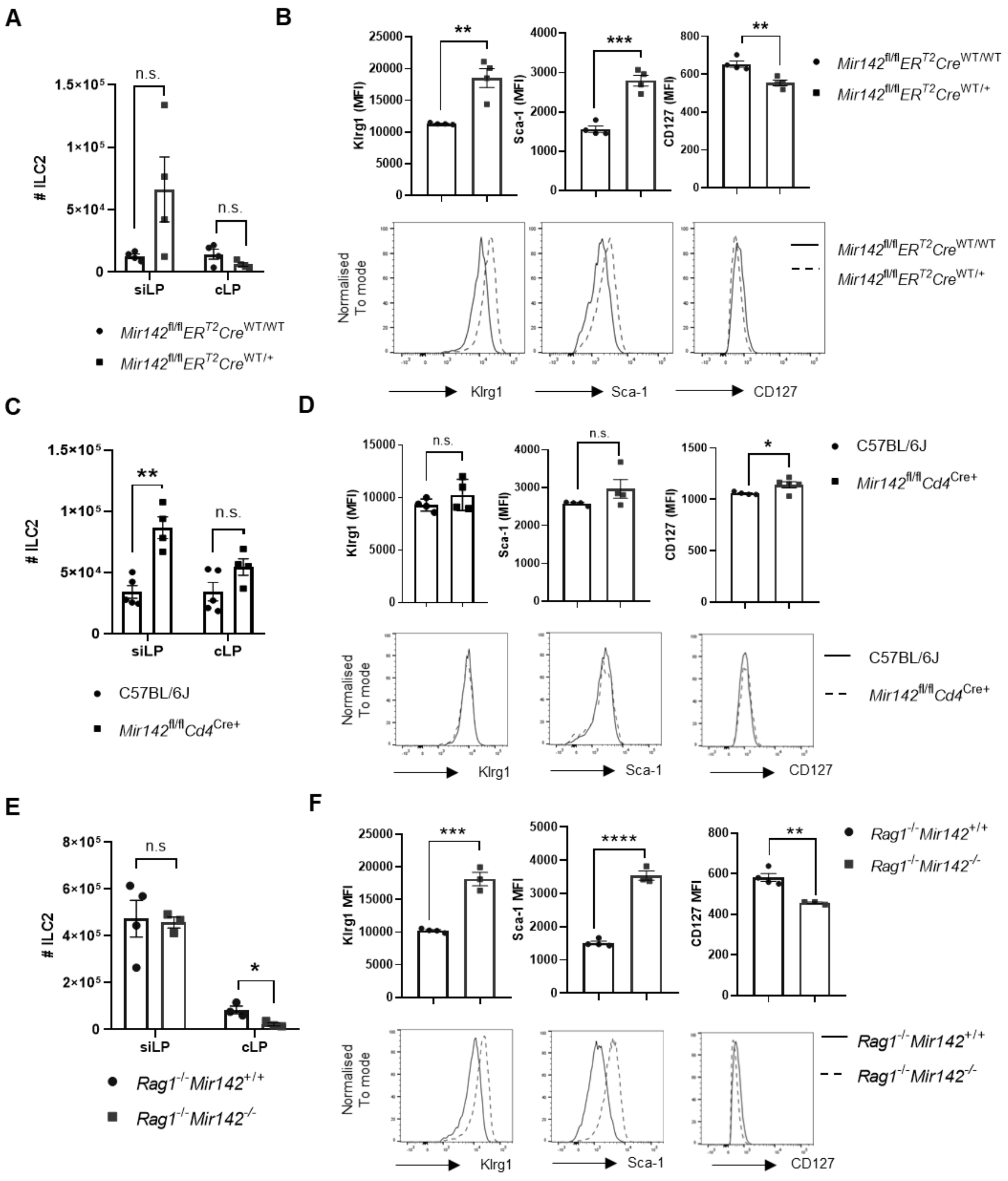

Supplemental Figure 4. Analysis of ILC2 numbers and phenotypes in temporally controlled, conditional, and constitutive models of *Mir142* deficiency. (Related to Figure 4). (A) Total number of ILC2 (CD45$^+$Lineage$^-$CD127$^+$NK1.1$^-$NKp46$^-$Rorγt$^-$Klrg1$^+$Sca-1$^+$) in the small intestinal lamina propria (siLP) and colonic lamina propria (cLP) of *Mir142*$^{F/F}$ER^{T2}Cre$^{WT/WT}$ and *Mir142*$^{F/F}$ER^{T2}Cre$^{WT/+}$ mice, 4 weeks post in vitro treatment with tamoxifen **(B)** Representative histograms and quantitation of mean fluorescence intensity (MFI) of Klrg1, Sca-1 and CD127 staining of siLP ILC2 from (A). **(C)** As for (A) but for C57BL/6J and *Mir142*$^{fl/fl}$*Cd4*$^{Cre+}$ mice. **(D)** As for (B) but for C57BL/6J and *Mir142*$^{fl/fl}$*Cd4*$^{Cre+}$ mice. **(E)** As for (A) but for *Rag1*$^{-/-}$*Mir142*$^{+/+}$ and *Rag1*$^{-/-}$*Mir142*$^{-/-}$ mice. **(F)** As for (B) but for *Rag1*$^{-/-}$*Mir142*$^{+/+}$ and *Rag1*$^{-/-}$*Mir142*$^{-/-}$ mice. All data is representative of at least 2 independent experiments with age and sex matched mice, n=3-5 animals per genotype. Bar graphs depict mean $\pm$ SEM. Unpaired t-test. *p <0.05, **p <0.01, ***p<0.001, ****p<0.0001. n.s., non-significant difference
